# Supplementary material for: Remarkable Single Atom Catalyst of Transition Metal (Fe, Co & Ni) Doped on C2N Surface for Hydrogen Dissociation Reaction
Source: Nanomaterials (Basel). 2022 Dec 21;13(1):29. doi: 10.3390/nano13010029 (PMC9823351; doi:10.3390/nano13010029)
Supplement: Supplementary file 1 [file nanomaterials-13-00029-s001.zip › nanomaterials-2093643-supplementary.pdf]

nanomaterials

Supplementary Materials

Remarkable Single Atom Catalyst of Transition Metal (Fe, Co & Ni) Doped on C_2N Surface for Hydrogen Dissociation Reaction

Ahmed Bilal Shah [1], Sehrish Sarfaraz [1], Muhammad Yar [1], Nadeem S. Sheikh [2], Hassan H. Hammud [3,*] and Khurshid Ayub [1,*]

[1] Department of Chemistry, COMSATS University Islamabad, Abbottabad Campus, Abbottabad 22060, Pakistan
[2] Chemical Sciences, Faculty of Science, Universiti Brunei Darussalam, Jalan Tungku Link, Gadong BE1410, Brunei
[3] Department of Chemistry, College of Science, King Faisal University, Al-Ahsa 31982, Saudi Arabia
* Correspondence: hhammoud@kfu.edu.sa (H.H.H.); khurshid@cuiatd.edu.pk (K.A.)

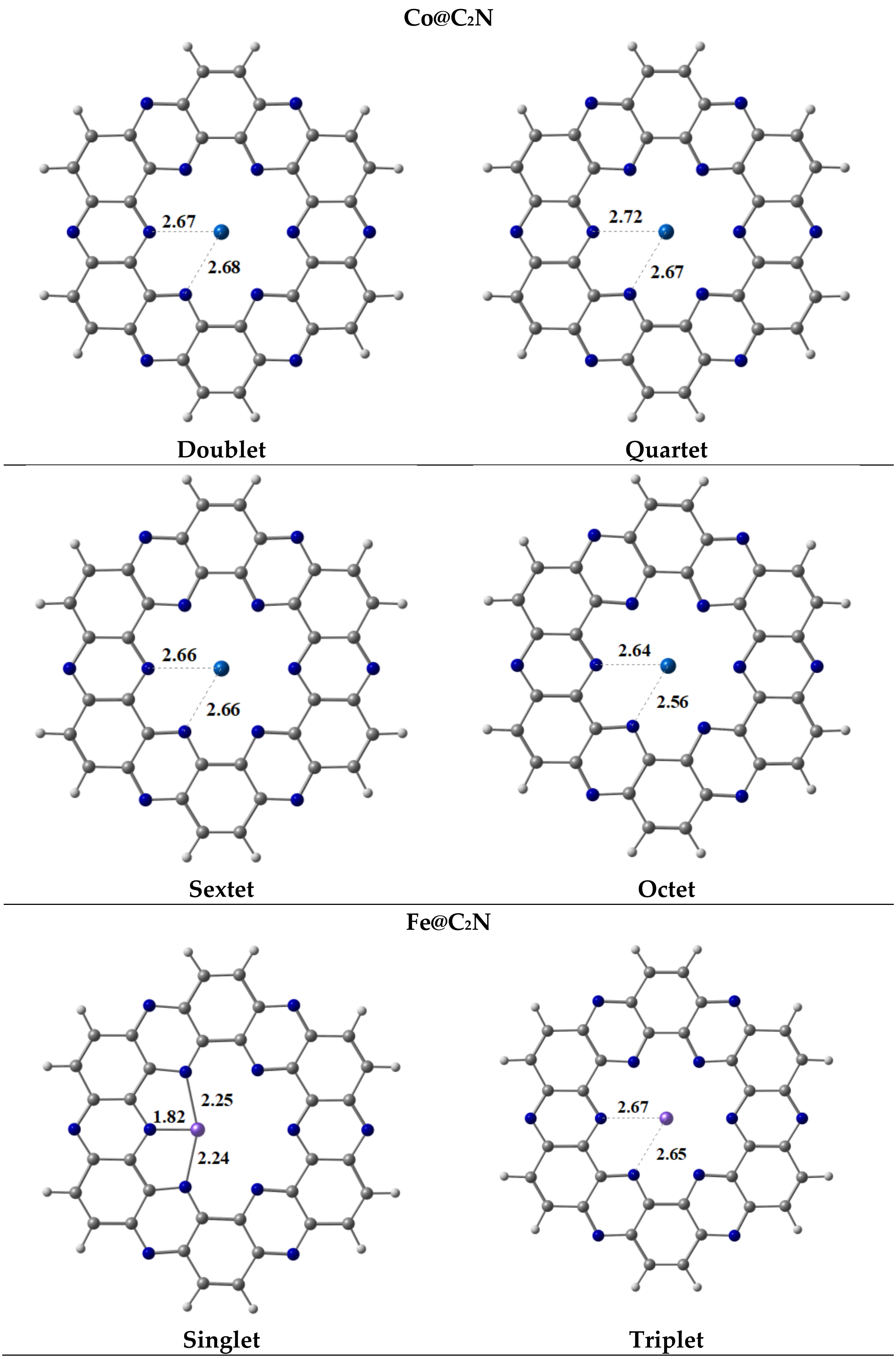
Co@C$_2$N
2.67
2.68
Doublet
2.72
2.67
Quartet
2.66
2.66
Sextet
2.64
2.56
Octet
Fe@C$_2$N
2.25
1.82
2.24
Singlet
2.67
2.65
Triplet

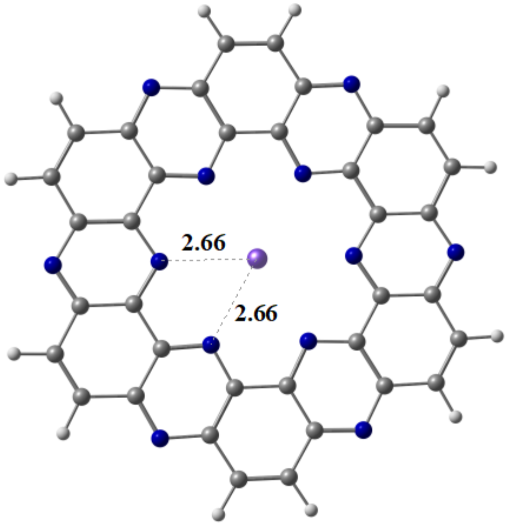

Quintet

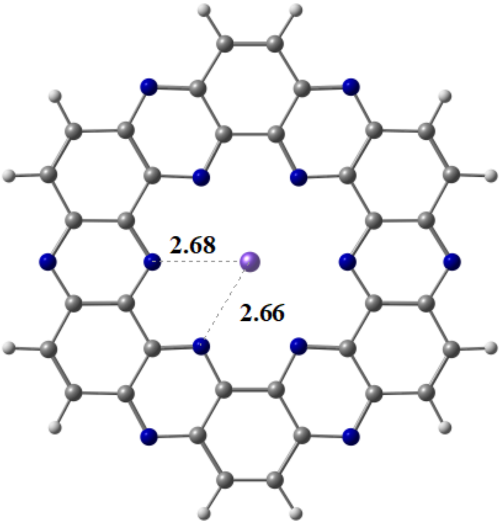

Septet

Ni@C_2N

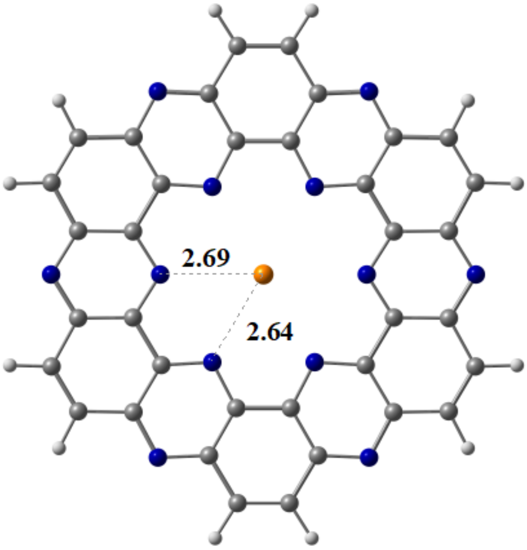

Singlet

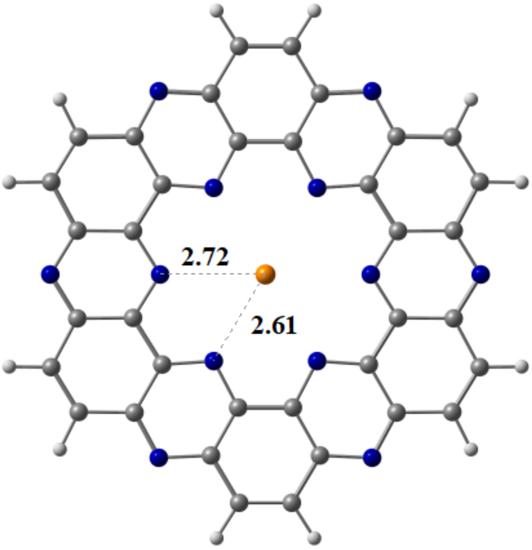

Triplet

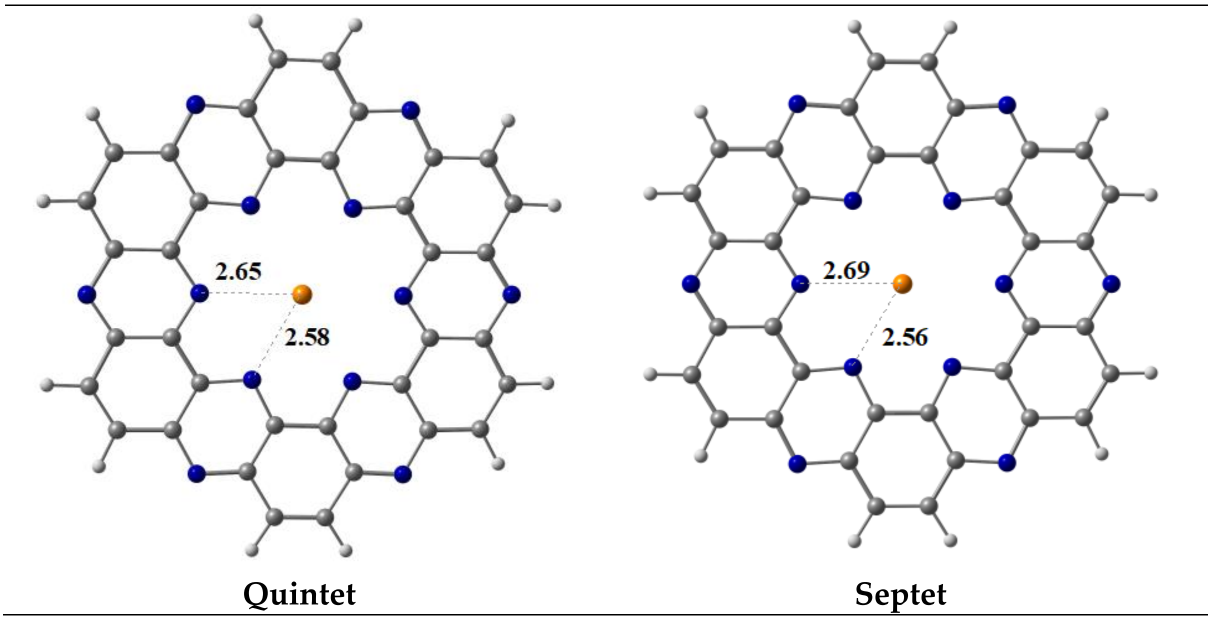

Figure S1. Optimized geometries of studied M@C_2N complexes at various possible spin states at M06-2X/6-31G(d,p) level of theory. Where grey color is for carbon, light grey for hydrogen, blue for nitrogen, orange for nickel, purple for iron and cobalt blue for cobalt atom.

Table S1. Relative stabilities of studied M@C_2N complexes at various possible spin states (all values are in eV).

M@C₂N	Doublet	Quartet	Sextet	Octet
**Co@C₂N**	**0.00**	0.35	0.63	4.48
**--**	**Singlet**	**Triplet**	**Quintet**	**Septet**
**Fe@C₂N**	1.32	0.13	0.29	**0.00**
**Ni@C₂N**	3.61	**0.00**	1.28	4.24

Table S2. Energies and lowest vibrational frequencies of reactants, products, and transition states over designed M@C_2N catalysts.

M@C₂N		Energies (au)	Frequency (cm^{-1})
	Reactant	-3419.17	27.96
**Co@C₂N**	TS	-3419.15	-433.35
	Product	-3419.18	32.87
	Reactant	-3300.20	27.68
**Fe@C₂N**	TS	-3300.19	-1166.19
	Product	-3300.20	34.83
	Reactant	-3544.71	31.58
**Co@C₂N**	TS	-3544.69	-424.51
	Product	-3544.70	32.97

Cartesian coordinates for designed catalysts

Co@C_2N

0 2

C 0.00000000 4.74502300 1.42119000

C 0.00000000 3.50322000 -0.72018500

C	0.00000000	5.96349200	-0.68373500
C	0.00000000	2.39272500	-5.50729300
C	0.00000000	3.60199900	-3.38808300
C	0.00000000	1.12603100	-3.37758100
C	0.00000000	1.13416400	-4.81070100
C	0.00000000	2.36925400	-2.66351900
C	0.00000000	3.56626000	-4.83368700
C	0.00000000	5.96349200	0.68373500
C	0.00000000	4.74502300	-1.42119000
C	0.00000000	3.50322000	0.72018500
N	0.00000000	4.77726900	-2.77109500
N	0.00000000	2.32009500	-1.32979300
N	0.00000000	0.00000000	-2.66501200
N	0.00000000	2.32009500	1.32979300
N	0.00000000	0.00000000	-5.51013400
N	0.00000000	4.77726900	2.77109500
C	0.00000000	-2.36925400	-2.66351900
C	0.00000000	-3.56626000	-4.83368700
C	0.00000000	-1.13416400	-4.81070100
C	0.00000000	-1.12603100	-3.37758100
C	0.00000000	-2.39272500	-5.50729300
C	0.00000000	-3.60199900	-3.38808300
N	0.00000000	-4.77726900	-2.77109500
N	0.00000000	-2.32009500	-1.32979300
C	0.00000000	2.36925400	2.66351900
C	0.00000000	3.56626000	4.83368700
C	0.00000000	1.13416400	4.81070100
C	0.00000000	1.12603100	3.37758100
C	0.00000000	2.39272500	5.50729300

C	0.00000000	3.60199900	3.38808300
N	0.00000000	0.00000000	5.51013400
N	0.00000000	0.00000000	2.66501200
C	0.00000000	-2.39272500	5.50729300
C	0.00000000	-3.60199900	3.38808300
C	0.00000000	-1.12603100	3.37758100
C	0.00000000	-4.74502300	-1.42119000
C	0.00000000	-3.50322000	0.72018500
C	0.00000000	-5.96349200	0.68373500
C	0.00000000	-5.96349200	-0.68373500
C	0.00000000	-4.74502300	1.42119000
C	0.00000000	-3.50322000	-0.72018500
C	0.00000000	-1.13416400	4.81070100
C	0.00000000	-2.36925400	2.66351900
C	0.00000000	-3.56626000	4.83368700
N	0.00000000	-2.32009500	1.32979300
N	0.00000000	-4.77726900	2.77109500
H	0.00000000	-2.35073900	-6.59096400
H	0.00000000	-4.52401000	-5.34156100
H	0.00000000	2.35073900	-6.59096400
H	0.00000000	4.52401000	-5.34156100
H	0.00000000	6.88468000	-1.25537600
H	0.00000000	6.88468000	1.25537600
H	0.00000000	4.52401000	5.34156100
H	0.00000000	2.35073900	6.59096400
H	0.00000000	-2.35073900	6.59096400
H	0.00000000	-4.52401000	5.34156100
H	0.00000000	-6.88468000	1.25537600
H	0.00000000	-6.88468000	-1.25537600

Co	0.00000000	0.00000000	0.00000000

Fe@C2N

0 7

C	0.00000000	4.76780100	1.41851200
C	0.00000000	3.53415700	-0.71605400
C	0.00000000	6.00401700	-0.67723500
C	0.00000000	2.37920400	-5.44020100
C	0.00000000	3.61816600	-3.37473300
C	0.00000000	1.13130500	-3.30656400
C	0.00000000	1.12825500	-4.72166900
C	0.00000000	2.37829500	-2.64588200
C	0.00000000	3.57589400	-4.80762400
C	0.00000000	6.00401700	0.67723500
C	0.00000000	4.76780100	-1.41851200
C	0.00000000	3.53415700	0.71605400
N	0.00000000	4.79524600	-2.75681700
N	0.00000000	2.34630600	-1.30753100
N	0.00000000	0.00000000	-2.53273700
N	0.00000000	2.34630600	1.30753100
N	0.00000000	0.00000000	-5.45030100
N	0.00000000	4.79524600	2.75681700
C	0.00000000	-2.37829500	-2.64588200
C	0.00000000	-3.57589400	-4.80762400
C	0.00000000	-1.12825500	-4.72166900
C	0.00000000	-1.13130500	-3.30656400
C	0.00000000	-2.37920400	-5.44020100
C	0.00000000	-3.61816600	-3.37473300
N	0.00000000	-4.79524600	-2.75681700
N	0.00000000	-2.34630600	-1.30753100

C	0.00000000	2.37829500	2.64588200
C	0.00000000	3.57589400	4.80762400
C	0.00000000	1.12825500	4.72166900
C	0.00000000	1.13130500	3.30656400
C	0.00000000	2.37920400	5.44020100
C	0.00000000	3.61816600	3.37473300
N	0.00000000	0.00000000	5.45030100
N	0.00000000	0.00000000	2.53273700
C	0.00000000	-2.37920400	5.44020100
C	0.00000000	-3.61816600	3.37473300
C	0.00000000	-1.13130500	3.30656400
C	0.00000000	-4.76780100	-1.41851200
C	0.00000000	-3.53415700	0.71605400
C	0.00000000	-6.00401700	0.67723500
C	0.00000000	-6.00401700	-0.67723500
C	0.00000000	-4.76780100	1.41851200
C	0.00000000	-3.53415700	-0.71605400
C	0.00000000	-1.12825500	4.72166900
C	0.00000000	-2.37829500	2.64588200
C	0.00000000	-3.57589400	4.80762400
N	0.00000000	-2.34630600	1.30753100
N	0.00000000	-4.79524600	2.75681700
H	0.00000000	-2.30042700	-6.52201000
H	0.00000000	-4.52132800	-5.33749400
H	0.00000000	2.30042700	-6.52201000
H	0.00000000	4.52132800	-5.33749400
H	0.00000000	6.92126400	-1.25534200
H	0.00000000	6.92126400	1.25534200
H	0.00000000	4.52132800	5.33749400

H	0.00000000	2.30042700	6.52201000
H	0.00000000	-2.30042700	6.52201000
H	0.00000000	-4.52132800	5.33749400
H	0.00000000	-6.92126400	1.25534200
H	0.00000000	-6.92126400	-1.25534200
Fe	0.00000000	0.00000000	0.00000000

Ni@C_2N

0 3

C	0.00000000	4.70459600	1.41706400
C	0.00000000	3.46662800	-0.71055400
C	0.00000000	5.94499100	-0.67510700
C	0.00000000	2.39863900	-5.52369500
C	0.00000000	3.58146900	-3.38667100
C	0.00000000	1.12213700	-3.40908000
C	0.00000000	1.13641600	-4.83857100
C	0.00000000	2.35318400	-2.66565300
C	0.00000000	3.56300700	-4.82488200
C	0.00000000	5.94499100	0.67510700
C	0.00000000	4.70459600	-1.41706400
C	0.00000000	3.46662800	0.71055400
N	0.00000000	4.76073400	-2.74770700
N	0.00000000	2.27564800	-1.33879100
N	0.00000000	0.00000000	-2.70543600
N	0.00000000	2.27564800	1.33879100
N	0.00000000	0.00000000	-5.54014400
N	0.00000000	4.76073400	2.74770700
C	0.00000000	-2.35318400	-2.66565300
C	0.00000000	-3.56300700	-4.82488200
C	0.00000000	-1.13641600	-4.83857100

C	0.00000000	-1.12213700	-3.40908000
C	0.00000000	-2.39863900	-5.52369500
C	0.00000000	-3.58146900	-3.38667100
N	0.00000000	-4.76073400	-2.74770700
N	0.00000000	-2.27564800	-1.33879100
C	0.00000000	2.35318400	2.66565300
C	0.00000000	3.56300700	4.82488200
C	0.00000000	1.13641600	4.83857100
C	0.00000000	1.12213700	3.40908000
C	0.00000000	2.39863900	5.52369500
C	0.00000000	3.58146900	3.38667100
N	0.00000000	0.00000000	5.54014400
N	0.00000000	0.00000000	2.70543600
C	0.00000000	-2.39863900	5.52369500
C	0.00000000	-3.58146900	3.38667100
C	0.00000000	-1.12213700	3.40908000
C	0.00000000	-4.70459600	-1.41706400
C	0.00000000	-3.46662800	0.71055400
C	0.00000000	-5.94499100	0.67510700
C	0.00000000	-5.94499100	-0.67510700
C	0.00000000	-4.70459600	1.41706400
C	0.00000000	-3.46662800	-0.71055400
C	0.00000000	-1.13641600	4.83857100
C	0.00000000	-2.35318400	2.66565300
C	0.00000000	-3.56300700	4.82488200
N	0.00000000	-2.27564800	1.33879100
N	0.00000000	-4.76073400	2.74770700
H	0.00000000	-2.37565200	-6.60760000
H	0.00000000	-4.52765100	-5.32063400

H	0.00000000	2.37565200	-6.60760000
H	0.00000000	4.52765100	-5.32063400
H	0.00000000	6.86036100	-1.25643100
H	0.00000000	6.86036100	1.25643100
H	0.00000000	4.52765100	5.32063400
H	0.00000000	2.37565200	6.60760000
H	0.00000000	-2.37565200	6.60760000
H	0.00000000	-4.52765100	5.32063400
H	0.00000000	-6.86036100	1.25643100
H	0.00000000	-6.86036100	-1.25643100
Ni	0.00000000	0.00000000	0.00000000

Cartesian coordinates of all computed geometries for hydrogen dissociation reaction

Co@C_2N

Reactant

0 2

C	-4.85717300	1.18926100	-0.13640800
C	-2.65243400	2.33455800	-0.01017900
C	-4.75868200	3.61450200	-0.09479500
C	0.80089300	5.88556700	0.09831100
C	-1.29033800	4.60731000	0.07019800
C	0.83204800	3.40687800	0.05201400
C	1.53891200	4.66237600	0.06247900
C	-0.61035600	3.35968800	0.06865300
C	-0.55251500	5.84157100	0.09929600
C	-5.49928800	2.47988400	-0.15414500
C	-3.30984500	3.58852800	-0.01948200
C	-3.43749100	1.18266800	-0.06261100
N	-2.64004100	4.73242700	0.03515700
N	-1.27929600	2.16159700	0.05746500

N	1.49870500	2.25550800	0.00823500
N	-2.75643800	0.02512600	-0.00695700
N	2.87584400	4.71960000	0.02831300
N	-5.56583100	0.05476300	-0.17341300
C	3.57015300	1.09634000	-0.06099200
C	5.67366600	2.37480200	-0.11266600
C	3.53137000	3.55990600	-0.01619200
C	2.82291200	2.33196800	-0.02221700
C	4.97903100	3.53794700	-0.06569400
C	4.98993000	1.09953400	-0.10811500
N	5.67960400	-0.04794900	-0.13573500
N	2.86763500	-0.02200800	-0.03885800
C	-3.46255500	-1.11942600	-0.06075600
C	-5.54387600	-2.37474700	-0.15134900
C	-3.37663800	-3.52473800	-0.00909900
C	-2.69830400	-2.28637200	-0.01051000
C	-4.82262300	-3.52426300	-0.08363100
C	-4.87836000	-1.10044100	-0.13704600
N	-2.72443600	-4.68533500	0.05278600
N	-1.31871700	-2.13526500	0.03129300
C	-0.65663500	-5.82809400	0.12058900
C	1.45438500	-4.68367900	0.05597300
C	-0.67047600	-3.34711900	0.06459400
C	4.96833400	-1.18286300	-0.11699600
C	2.77633700	-2.37475600	-0.04008600
C	4.91061700	-3.61912500	-0.08452700
C	5.62834000	-2.46978400	-0.12833300
C	3.46351100	-3.61427000	-0.03343900
C	3.54806800	-1.15370900	-0.07120400

C	-1.37583200	-4.58124500	0.08473800
C	0.76711600	-3.41624700	0.03896400
C	0.69522900	-5.89415300	0.10745500
N	1.45285000	-2.27415200	-0.01013400
N	2.78828800	-4.76385100	0.01653800
H	5.48032700	4.50016300	-0.06494700
H	6.75737000	2.35651400	-0.15058100
H	1.36028800	6.81358800	0.11275400
H	-1.15570000	6.74290800	0.11295700
H	-5.21620700	4.59777900	-0.10481800
H	-6.58207200	2.50185700	-0.21270600
H	-6.62693900	-2.37824700	-0.20795600
H	-5.29944100	-4.49838900	-0.08514800
H	-1.27411900	-6.71939300	0.14747700
H	1.23928300	-6.83127000	0.12613700
H	5.39294800	-4.59106000	-0.08631800
H	6.71210900	-2.47191500	-0.16637600
H	0.89262300	0.00088400	0.94782300
H	0.37907800	-0.01343200	1.51248000
Co	-0.85893700	-0.01862100	0.15813500

Co@C_2N

Transition state

0 2

C	4.79930000	-1.14561600	-0.01286900
C	2.65575000	-2.35265700	-0.01085300
C	4.78862200	-3.58720200	-0.02874400
C	-0.73492400	-5.94015000	-0.02841800
C	1.35426600	-4.68356500	-0.03039700

C	-0.79981200	-3.46370800	-0.03519700
C	-1.48647100	-4.71871200	-0.02592300
C	0.65365200	-3.43014800	-0.02479200
C	0.62015000	-5.92091900	-0.03314500
C	5.47763300	-2.41801800	-0.02407700
C	3.34891600	-3.59577800	-0.02329500
C	3.39164900	-1.15166300	-0.00604300
N	2.69067300	-4.75097300	-0.02930400
N	1.30163000	-2.25901800	-0.00591500
N	-1.43761800	-2.31052100	-0.04799300
N	2.66289700	-0.00309900	0.00550400
N	-2.82663900	-4.77603300	-0.02435700
N	5.52067600	-0.00402400	-0.01077500
C	-3.48669600	-1.14163100	0.01426800
C	-5.58768600	-2.41143200	0.00150800
C	-3.48133600	-3.61782500	-0.01481000
C	-2.77861500	-2.36730200	-0.01750100
C	-4.91813100	-3.59383500	-0.00690000
C	-4.90362300	-1.13855000	0.00901500
N	-5.62201800	-0.00438100	0.01319800
N	-2.76068900	0.00240900	0.04067300
C	3.39557900	1.14521200	0.00263700
C	5.48375000	2.40952300	-0.00494700
C	3.35129200	3.58431200	-0.00469800
C	2.65989600	2.34100000	0.00112600
C	4.79267300	3.57645000	-0.00484900
C	4.80468700	1.13542300	-0.00364400
N	2.69383300	4.73888800	-0.01251900
N	1.30136800	2.24292800	-0.00056100

C	0.62513300	5.91075300	-0.03013400
C	-1.48447700	4.71959800	-0.03543400
C	0.65507200	3.41922200	-0.02423100
C	-4.90034200	1.14249000	0.00007700
C	-2.77588700	2.37218100	-0.02891200
C	-4.91125700	3.59766000	-0.02703600
C	-5.57961000	2.40786800	-0.01636000
C	-3.48235000	3.62046300	-0.03030000
C	-3.48593500	1.13588200	0.00658600
C	1.35679100	4.66866700	-0.02306000
C	-0.79496900	3.46323600	-0.04292700
C	-0.72735000	5.94153800	-0.03371100
N	-1.44150400	2.31368700	-0.05776300
N	-2.81744100	4.78070300	-0.03943000
H	-5.43392400	-4.54779600	-0.00844700
H	-6.67212500	-2.37390900	0.00441200
H	-1.29902000	-6.86642900	-0.02790600
H	1.21231800	-6.82871800	-0.03620400
H	5.28533100	-4.55091700	-0.03814800
H	6.56190800	-2.39243400	-0.02954600
H	6.56800500	2.38495400	-0.00832400
H	5.28740800	4.54129900	-0.00815800
H	1.22424600	6.81428000	-0.02922300
H	-1.28659700	6.87049900	-0.03598800
H	-5.43056300	4.54926300	-0.03271300
H	-6.66417600	2.37147900	-0.01567900
H	-1.28166000	0.00712500	0.70850600
H	-0.61145000	0.01417600	1.22737400
Co	0.75229900	0.02728000	0.11491400

$Co@C_2N$

Product

0 2

C	-4.85247200	1.02865500	-0.09731400
C	-2.72748200	2.28198800	-0.00433200
C	-4.88359500	3.47519200	-0.07777200
C	0.59297600	5.94664700	0.03207200
C	-1.46298100	4.63105300	0.01512700
C	0.71710200	3.46246700	-0.00143100
C	1.37601300	4.73628200	0.00922200
C	-0.74054000	3.39839800	0.01873100
C	-0.75587200	5.89038300	0.03070000
C	-5.55143700	2.29361300	-0.11446400
C	-3.44319700	3.51476100	-0.02688400
C	-3.44553000	1.06816700	-0.03585100
N	-2.80293500	4.67783100	-0.00438900
N	-1.37166600	2.21533400	0.03107700
N	1.40141700	2.33271700	-0.04548800
N	-2.69764800	-0.06353600	-0.00691200
N	2.70731700	4.83798200	-0.02613100
N	-5.54052700	-0.12816900	-0.13531600
C	3.50122700	1.24797000	-0.02294900
C	5.54675300	2.54738900	-0.11922000
C	3.40744400	3.69951300	-0.04586800
C	2.73681100	2.44120400	-0.03669800
C	4.84214100	3.71239900	-0.09265700
C	4.90253700	1.25785200	-0.08397000
N	5.64910200	0.12450700	-0.09565200

N	2.83997400	0.05956800	0.04771100
C	-3.38758100	-1.22804700	-0.04858200
C	-5.43194400	-2.54896100	-0.13995600
C	-3.27631000	-3.67576900	-0.05158900
C	-2.61354100	-2.41452500	-0.02242300
C	-4.71303400	-3.70259600	-0.10912800
C	-4.79201000	-1.25779500	-0.11004200
N	-2.58163700	-4.81224400	-0.02612300
N	-1.26595300	-2.29090300	0.01959200
C	-0.48390300	-5.93305700	0.02764800
C	1.59364900	-4.67819700	0.02368300
C	-0.58323300	-3.43997900	0.01683500
C	4.95669000	-1.04241200	-0.07292900
C	2.84880200	-2.32450300	-0.01419700
C	5.00931800	-3.49751400	-0.06692800
C	5.65938400	-2.30087300	-0.09927200
C	3.57608200	-3.55060700	-0.02186300
C	3.55614900	-1.09702000	-0.00957300
C	-1.25005600	-4.70707900	0.00597400
C	0.87781000	-3.43581600	0.01159900
C	0.86541600	-5.92399900	0.03978200
N	1.50938100	-2.27675300	-0.02252500
N	2.92764000	-4.72014500	-0.00316600
H	5.33318200	4.67884500	-0.10760200
H	6.63078000	2.54362000	-0.15846800
H	1.13625000	6.88471400	0.04053100
H	-1.37209800	6.78247000	0.03832000
H	-5.40009800	4.42835100	-0.09084800
H	-6.63453300	2.25331200	-0.15802400

H	-6.51569800	-2.55640100	-0.18603900
H	-5.18732300	-4.67718700	-0.12938400
H	-1.05675900	-6.85356100	0.02974700
H	1.45246400	-6.83541900	0.05183800
H	5.54498500	-4.44000000	-0.07786700
H	6.74209800	-2.24739800	-0.13873500
H	1.84774500	0.03814300	0.31588000
H	0.40376800	0.06523100	1.39547300
Co	-0.79168600	0.03669800	0.32642800

Fe@C_2N

Reactant

0 7

C	1.36018200	-4.63753400	-0.00443400
C	-0.76589100	-3.44447200	0.00388700
C	-0.72770500	-5.92095900	-0.01199400
C	-5.60680300	-2.41912500	-0.04293100
C	-3.45764900	-3.58958300	-0.01796100
C	-3.50461600	-1.12822300	-0.01226000
C	-4.92691500	-1.14477700	-0.03390600
C	-2.74907800	-2.35825800	-0.00767200
C	-4.90309600	-3.57690200	-0.03404800
C	0.62653100	-5.87661600	-0.01082700
C	-1.47299500	-4.69986600	-0.00793900
C	0.66001400	-3.40495100	0.01000900
N	-2.80601200	-4.75492300	-0.01761900
N	-1.42356200	-2.27834200	0.00306200
N	-2.81070900	-0.00249200	0.00321900

N	1.30282100	-2.19324400	0.02467100
N	-5.62815100	-0.00530900	-0.04230900
N	2.70810900	-4.73244800	-0.01432100
C	-2.75392900	2.35335100	-0.00797500
C	-4.91027300	3.56780600	-0.03464100
C	-4.92921800	1.13550500	-0.03413900
C	-3.50694200	1.12179200	-0.01247100
C	-5.61159700	2.40861500	-0.04340900
C	-3.46477000	3.58337700	-0.01841100
N	-2.81532600	4.74981900	-0.01803600
N	-1.42832900	2.27596700	0.00289600
C	2.67351600	-2.32386300	0.00248100
C	4.80092300	-3.55885800	-0.03906100
C	4.86061500	-1.13820700	-0.03295300
C	3.43248100	-1.14201000	-0.00611700
C	5.52526900	-2.40876700	-0.04732500
C	3.35688600	-3.56071800	-0.01703600
N	5.55461200	0.00543800	-0.04439000
N	2.73529400	0.00252600	0.00968400
C	5.52056400	2.41943400	-0.04695400
C	3.34982000	3.56706400	-0.01663300
C	3.43008200	1.14849000	-0.00596600
C	-1.48208000	4.69726300	-0.00815600
C	0.65337400	3.40608600	0.01012700
C	0.61527900	5.87766200	-0.01068500
C	-0.73909200	5.91965500	-0.01208200
C	1.35106800	4.63997400	-0.00419400
C	-0.77275500	3.44317700	0.00380200
C	4.85836100	1.14740100	-0.03278000

C	2.66882400	2.32878000	0.00274400
C	4.79403600	3.56799500	-0.03857200
N	1.29832100	2.19558100	0.02483600
N	2.69905700	4.73738800	-0.01390400
H	-6.69554400	2.39076400	-0.05755000
H	-5.40403600	4.53365700	-0.04212900
H	-6.69077800	-2.40346000	-0.05699700
H	-5.39490400	-4.54375200	-0.04136900
H	-1.28473200	-6.85054300	-0.01843000
H	1.22955400	-6.77823000	-0.01747400
H	5.27741600	-4.53299600	-0.05039900
H	6.60941400	-2.40951300	-0.06551000
H	6.60470700	2.42225400	-0.06510000
H	5.26854400	4.54310700	-0.04977400
H	1.21671000	6.78034100	-0.01723800
H	-1.29773100	6.84826400	-0.01860700
H	-0.91276300	0.00307600	1.41698300
H	-0.32362400	-0.00055500	1.88774300
Fe	0.63770600	-0.00002200	0.05747000

Fe@C_2N

Transition state

0 7

C	2.41374900	-4.23958700	0.00711100
C	0.06208200	-3.53798700	0.00564300
C	0.65724400	-5.93651700	-0.00528700
C	-4.88039300	-3.61399300	-0.05780600
C	-2.51986900	-4.25824600	-0.03815500
C	-3.13222200	-1.86163000	-0.05679900

C	-4.51671100	-2.21299500	-0.05658300
C	-2.10792500	-2.89049700	-0.04106000
C	-3.93339900	-4.57835000	-0.04980700
C	1.97523900	-5.60426800	-0.00136600
C	-0.33926900	-4.90974700	-0.00717700
C	1.45662000	-3.20099500	0.02417500
N	-1.63542100	-5.25056600	-0.02420600
N	-0.82721700	-2.53472800	-0.01283000
N	-2.73339300	-0.60584300	-0.05733500
N	1.80993300	-1.89412400	0.04448500
N	-5.47080500	-1.28375500	-0.06674400
N	3.75615300	-3.99854400	-0.00539400
C	-3.25195000	1.68726400	0.03065800
C	-5.59691500	2.36777600	-0.05555200
C	-5.07402600	0.00067900	-0.04702000
C	-3.68503100	0.32415100	-0.02176700
C	-6.02424900	1.07021400	-0.06377400
C	-4.20766200	2.71700100	-0.01146000
N	-3.82961500	4.02840400	-0.00214700
N	-1.92995400	1.92152300	0.11174000
C	3.16070900	-1.67027800	0.01424000
C	5.52267200	-2.36845000	-0.04321300
C	4.97359200	0.00782700	-0.04624100
C	3.58689400	-0.33827300	-0.00251300
C	5.93249400	-1.07974900	-0.06100300
C	4.11326100	-2.72425500	-0.01083200
N	5.37840300	1.26511000	-0.07342200
N	2.62669600	0.60950300	0.01751200
C	4.78735700	3.60501700	-0.09571600

C	2.43921900	4.25435800	-0.05004000
C	3.04066500	1.88813400	-0.01948900
C	-2.52656900	4.26444200	0.01601500
C	-0.16283100	3.51495500	0.03357200
C	-0.75248700	5.93512100	-0.02161300
C	-2.06844300	5.64012100	-0.01154600
C	0.25720400	4.89378500	-0.01011700
C	-1.54031800	3.22602300	0.06614400
C	4.41521900	2.22402300	-0.06388500
C	2.03245400	2.90105200	-0.01403600
C	3.83387100	4.57832700	-0.08870400
N	0.74477300	2.50097400	0.02406600
N	1.52527900	5.25569600	-0.04994700
H	-7.07673100	0.81244900	-0.08720500
H	-6.30255600	3.19194600	-0.07717000
H	-5.93962300	-3.84714100	-0.06396800
H	-4.18726200	-5.63278700	-0.04916000
H	0.31545000	-6.96491400	-0.01197300
H	2.75444400	-6.35913600	-0.00729300
H	6.22611100	-3.19398600	-0.05625900
H	6.98249400	-0.80961400	-0.08870000
H	5.84638900	3.83479500	-0.12528000
H	4.09073400	5.63163000	-0.11264800
H	-0.38736700	6.95605200	-0.04824200
H	-2.83493600	6.40739100	-0.03245200
H	-1.12332800	0.97599600	0.77402900
H	-0.64426800	0.40121800	1.33036400
Fe	0.62826900	-0.07485300	0.17098200

Fe@C_2N

Product

0 7

C	-4.29945100	2.42307400	-0.08087000
C	-1.90778400	3.01528100	0.01258100
C	-3.63089700	4.77446800	-0.08708600
C	2.32554800	5.55986700	-0.02377100
C	-0.03152600	4.91992400	-0.00816500
C	1.71662800	3.15007600	-0.00834500
C	2.72335600	4.16946600	-0.02978600
C	0.30554500	3.52722700	0.00961100
C	1.02266700	5.91347300	-0.01416900
C	-4.60587100	3.82163000	-0.11615700
C	-2.25081400	4.39795800	-0.03112700
C	-2.94955500	2.02805100	0.00261400
N	-1.29392100	5.33787900	-0.02778500
N	-0.63487700	2.58770800	0.03622800
N	2.03019100	1.86879900	-0.01758200
N	-2.58719400	0.73478600	0.05489000
N	4.02219300	3.87071400	-0.07798300
N	-5.31533800	1.50801500	-0.12898800
C	3.70670800	0.20339300	0.01195600
C	6.04256500	0.82710100	-0.16778900
C	4.35200900	2.57178700	-0.07742100
C	3.33749700	1.57187100	-0.02130000
C	5.72115200	2.15205900	-0.15146900
C	5.04312000	-0.20732100	-0.08998100
N	5.40690900	-1.51585100	-0.10450100
N	2.72204600	-0.73020300	0.12869500

C	-3.59726300	-0.17582700	-0.01538900
C	-5.96322500	-0.81457800	-0.17357000
C	-4.23909600	-2.54632200	-0.09019400
C	-3.23647900	-1.53097500	-0.00950400
C	-5.62502600	-2.12495500	-0.16542100
C	-4.95711700	0.23194700	-0.10613700
N	-3.92739600	-3.82954200	-0.09787300
N	-1.91354700	-1.83560900	0.06076900
C	-2.23193000	-5.52931200	-0.06899100
C	0.11966000	-4.93280400	-0.00996300
C	-1.60534100	-3.14201700	0.02112800
C	4.39694700	-2.41726800	-0.05964300
C	1.99699500	-3.01425400	0.03744500
C	3.72656900	-4.77403900	-0.08715600
C	4.70020600	-3.82721600	-0.11477000
C	2.33859800	-4.40336100	-0.02011100
C	3.04406500	-2.05364200	0.04896900
C	-2.60735800	-4.14062200	-0.04796600
C	-0.21084000	-3.54188800	0.03038600
C	-0.93248800	-5.91364700	-0.04700900
N	0.72214500	-2.59379800	0.05016600
N	1.39355600	-5.34328600	-0.03746800
H	6.48049900	2.92437900	-0.19960900
H	7.07412900	0.49795100	-0.23289500
H	3.12241300	6.29538100	-0.03512500
H	0.70581800	6.95037800	-0.01692400
H	-3.85851100	5.83363000	-0.11358800
H	-5.65605600	4.08844600	-0.16936900
H	-6.99692100	-0.49170100	-0.23485200

H	-6.37117100	-2.91016900	-0.21944700
H	-3.04263000	-6.24853200	-0.10850900
H	-0.63539100	-6.95579000	-0.06783300
H	3.95381700	-5.83334500	-0.12705700
H	5.74992600	-4.09247600	-0.17924100
H	1.79876300	-0.44084900	0.48433700
H	0.55752100	-0.04139700	1.55154800
Fe	-0.68008600	-0.03864600	0.43423200

Ni@C_2N

Reactant

0 3

C	4.92262800	-0.57466300	-0.02952800
C	2.92792500	-2.01507600	0.02864000
C	5.16908900	-2.99189100	0.00398800
C	-0.06889000	-5.96442100	-0.00923500
C	1.87249100	-4.46312400	0.02602300
C	-0.37866100	-3.50732700	0.00466900
C	-0.94566800	-4.83227500	-0.01620400
C	1.03505700	-3.31540400	0.03019900
C	1.27301100	-5.77384400	0.01121400
C	5.73854700	-1.75428700	-0.02141800
C	3.74065300	-3.16799900	0.02407500
C	3.52299300	-0.75610500	-0.00409700
N	3.22044400	-4.40724800	0.03274600
N	1.54334100	-2.04763800	0.05425100
N	-1.16077200	-2.41869600	-0.00552700
N	2.65581400	0.30381200	-0.02556400
N	-2.26371900	-5.02884700	-0.03980800

N	5.48550000	0.65416700	-0.06008200
C	-3.36106000	-1.49398400	-0.03631600
C	-5.30706400	-3.01569200	-0.06760400
C	-3.03938600	-3.93881000	-0.04632000
C	-2.47103500	-2.63688000	-0.03203100
C	-4.47585400	-4.08681000	-0.06637900
C	-4.77613700	-1.67415000	-0.04972700
N	-5.60481400	-0.62259700	-0.04441300
N	-2.80430400	-0.29527800	-0.02076500
C	3.22854600	1.52835200	-0.03969400
C	5.15920900	3.03906300	-0.07739500
C	2.90377100	3.95762700	-0.04078200
C	2.35629000	2.63313700	-0.02808300
C	4.33934600	4.11704300	-0.06800400
C	4.63851300	1.69015200	-0.06016700
N	2.12540100	5.02698800	-0.02612400
N	1.02260500	2.38701600	-0.00213100
C	-0.07193600	5.94996800	0.00897200
C	-2.02561600	4.52827500	0.02161300
C	0.25107000	3.48900200	0.00740700
C	-5.04111900	0.59046000	-0.02314700
C	-3.03624700	2.05652100	0.00938500
C	-5.31389500	3.01609200	0.00152600
C	-5.86650900	1.77964900	-0.01497700
C	-3.87772900	3.20044900	0.01147800
C	-3.63017000	0.74023600	-0.01096300
C	0.79639800	4.80183200	-0.00232900
C	-1.19360400	3.36296900	0.02009000
C	-1.42080100	5.83098100	0.02095700

N	-1.71083200	2.14139400	0.02062200
N	-3.36101700	4.43026800	0.02152600
H	-4.85666700	-5.10260900	-0.07878800
H	-6.38577300	-3.12323500	-0.08018400
H	-0.51999000	-6.94980300	-0.02201000
H	1.96786000	-6.60698000	0.01390800
H	5.76880900	-3.89556400	0.00571900
H	6.81432200	-1.61825900	-0.03841800
H	6.23901800	3.14199200	-0.09574300
H	4.71768800	5.13284400	-0.07803400
H	0.41860100	6.91732600	0.00374800
H	-2.08248400	6.68929600	0.02596700
H	-5.92176400	3.91429300	0.00560000
H	-6.94121000	1.63363100	-0.02450400
H	-0.45362300	0.30209300	1.42209500
H	-0.34837500	-0.44175100	1.51310700
Ni	0.78946900	-0.10539600	0.04212100

Ni@C_2N

Transition state

0 3

C	-4.88368200	0.62319600	0.04563800
C	-2.89242700	2.06201800	0.00764300
C	-5.14792700	3.05265700	0.02073300
C	0.09830900	5.98208700	-0.10353800
C	-1.85214300	4.52428400	-0.06499100
C	0.41833000	3.52919100	-0.07249400
C	0.97048600	4.84724800	-0.08328800
C	-1.02247200	3.35477300	-0.05683900
C	-1.25088200	5.82765700	-0.09591600

C	-5.70022900	1.81446300	0.04352100
C	-3.71699200	3.22322600	-0.00294500
C	-3.48342500	0.78317600	0.03796700
N	-3.19107700	4.44239200	-0.04004000
N	-1.54016200	2.12246400	-0.02129900
N	1.16660500	2.44198600	-0.05844500
N	-2.62802100	-0.27668400	0.04528100
N	2.30187500	5.03964100	-0.07860900
N	-5.47283100	-0.58652100	0.04919900
C	3.33095400	1.49519900	0.04502900
C	5.29138400	2.98229000	0.01244800
C	3.06569800	3.95503200	-0.04112900
C	2.49740800	2.63575000	-0.01918500
C	4.49975300	4.08296000	-0.02469000
C	4.74413900	1.64278000	0.04053000
N	5.57766700	0.59737500	0.06076800
N	2.73503300	0.27781700	0.09402500
C	-3.23202100	-1.49755100	0.01957700
C	-5.17897300	-2.97747200	0.00983000
C	-2.94326500	-3.92905700	-0.03603300
C	-2.37941500	-2.62161700	-0.01895900
C	-4.37590100	-4.06972300	-0.02047200
C	-4.63336400	-1.64186300	0.02804000
N	-2.17096900	-5.01317500	-0.06870400
N	-1.04418700	-2.39585000	-0.03975700
C	0.01253600	-5.96763000	-0.09986600
C	1.98333100	-4.55164400	-0.06378300
C	-0.28345000	-3.49549200	-0.06456500
C	4.98535300	-0.62467600	0.05115500

C	3.00973900	-2.07819900	0.00688900
C	5.26603200	-3.06256500	0.00844200
C	5.79936300	-1.80618200	0.03287900
C	3.84844000	-3.24039500	-0.00859900
C	3.57930100	-0.76843900	0.05877200
C	-0.84982900	-4.81041300	-0.07853100
C	1.16259300	-3.37899000	-0.06145700
C	1.35965800	-5.84988100	-0.09163300
N	1.67854200	-2.16515000	-0.03849400
N	3.31393000	-4.46794700	-0.04626000
H	4.90867200	5.08714200	-0.04372400
H	6.37358800	3.05941000	0.02065100
H	0.56413000	6.96132200	-0.12144300
H	-1.92958400	6.67256600	-0.10612100
H	-5.75031000	3.95402700	0.01454200
H	-6.77462100	1.66698200	0.05597400
H	-6.26024900	-3.06071700	0.01948400
H	-4.77143400	-5.07896300	-0.03544000
H	-0.48293300	-6.93182500	-0.11660600
H	2.01599400	-6.71297300	-0.10228100
H	5.88612400	-3.95147300	-0.00296000
H	6.87332700	-1.65095500	0.03880300
H	1.22998200	0.12329200	0.79247700
H	0.56518400	0.05453800	1.30063900
Ni	-0.71825600	-0.02085700	0.11147400

Ni@C_2N

Product

0 3

C	-3.35781500	-3.59638600	-0.03923000

C	-3.41275500	-1.14746100	-0.03149500
C	-5.48946700	-2.42158800	-0.12781500
C	-4.79794500	3.58855900	-0.09410300
C	-4.81914300	1.14236200	-0.09901800
C	-2.66787300	2.34881200	-0.01260900
C	-3.35853600	3.59564100	-0.03937900
C	-3.41303800	1.14676700	-0.03159600
C	-5.49014500	2.42044900	-0.12794500
C	-4.79706800	-3.58969200	-0.09394000
C	-4.81872900	-1.14356200	-0.09896700
C	-2.66737000	-2.34957100	-0.01242500
N	-5.53736100	-0.00052600	-0.12845200
N	-2.69523100	-0.00029800	0.02884400
N	-1.31702600	2.25497000	0.01378200
N	-1.31653500	-2.25548000	0.01394800
N	-2.69260300	4.74635400	-0.02174500
N	-2.69146900	-4.74708900	-0.02164800
C	0.79804800	3.45128400	-0.00357200
C	0.73026200	5.93797200	0.02283100
C	-1.35587400	4.67144800	0.00257700
C	-0.66147300	3.42195100	0.00886600
C	-0.61940300	5.91453400	0.01686700
C	1.48588000	4.70949700	0.00686900
N	2.81914100	4.78094600	-0.01991100
N	1.45597800	2.30608100	-0.03798600
C	-0.66072300	-3.42213800	0.00886100
C	-0.61814700	-5.91486800	0.01672400
C	1.48684100	-4.70919300	0.00676100
C	0.79880800	-3.45119000	-0.00358700

C	0.73150600	-5.93787100	0.02262300
C	-1.35500800	-4.67195500	0.00256300
N	2.82017100	-4.78038700	-0.01995900
N	1.45646100	-2.30581700	-0.03794800
C	4.92858700	-3.60517300	-0.07460400
C	4.93103700	-1.14981100	-0.07116000
C	2.79415600	-2.38369300	-0.02771500
C	3.49311900	3.62653300	-0.03529700
C	3.52922500	1.17344100	-0.01663200
C	5.60509100	2.42499500	-0.09938700
C	4.92780200	3.60617500	-0.07469100
C	4.93072400	1.15087600	-0.07121900
C	2.79363300	2.38426300	-0.02775100
C	3.49387100	-3.62587300	-0.03525600
C	3.52944900	-1.17275100	-0.01660600
C	5.60562800	-2.42388800	-0.09928400
N	2.83996900	0.00027500	0.04195400
N	5.65050100	0.00051700	-0.08529200
H	-1.21388900	6.82126000	0.02010100
H	1.29573800	6.86289700	0.03079100
H	-5.29385400	4.55241800	-0.11421200
H	-6.57368100	2.40251200	-0.17583700
H	-6.57300400	-2.40389000	-0.17573800
H	-5.29283800	-4.55361300	-0.11405600
H	-1.21232300	-6.82179300	0.01991100
H	1.29723200	-6.86264900	0.03049700
H	5.44236600	-4.55976400	-0.08643500
H	6.68943100	-2.39444400	-0.13331200
H	6.68890000	2.39578100	-0.13346100

H	5.44137800	4.56087100	-0.08653900
H	1.84378900	0.00018000	0.28799000
H	0.36696400	0.00026600	1.38710600
Ni	-0.78032500	0.00034900	0.33080500
